# Far-red/near-infrared fluorescence light-up probes for specific *in vitro* and *in vivo* imaging of a tumour-related protein

**DOI:** 10.1038/srep23190

**Published:** 2016-03-17

**Authors:** Chao Chen, Yongquan Hua, Yawen Hu, Yuan Fang, Shenglu Ji, Zhimou Yang, Caiwen Ou, Deling Kong, Dan Ding

**Affiliations:** 1 State Key Laboratory of Medicinal Chemical Biology, Key Laboratory of Bioactive Materials, Ministry of Education, College of Life Sciences, and Collaborative Innovation Center of Chemical Science and Engineering (Tianjin), Nankai University, Tianjin, 300071, P. R. China; 2 Department of Cardiology, Zhujiang Hospital of Southern Medical University, Southern Medical University, Guangzhou 510280, P. R. China

## Abstract

As lysosomal protein transmembrane 4 beta (LAPTM4B) is an important biomarker for many solid tumours, development of small-molecule fluorescence light-up probes for detection and imaging of LAPTM4B proteins is particularly valuable. In this work, we reported the design and synthesis of a far-red/near-infrared (FR/NIR) fluorescence light-up probe DBT-2EEGIHGHHIISVG, which could specifically visualize LAPTM4B proteins in cancer cells and tumour-bearing live mice. DBT-2EEGIHGHHIISVG was synthesized by the conjugation of two LAPTM4B-binding peptide ligands (EEGIHGHHIISVG) with one environment-sensitive fluorogen, 4,7-di(thiophen-2-yl)-2,1,3-benzothiadiazole (DBT). Owing to the intramolecular charge transfer character of DBT, DBT-2EEGIHGHHIISVG is weakly emissive in aqueous solution, but switches to fluoresce upon LAPTM4B proteins specifically bind to the peptide ligand of the probe, which provide the DBT with hydrophobic microenvironment, greatly reducing its charge transfer effect with water. It is found that DBT-2EEGIHGHHIISVG can achieve targeted imaging of LAPTM4B proteins in HepG2 cancer cells and visualize LAPTM4B protein-expressed tumour tissues of live mice in a selective and high-contrast manner.

Selective detection and sensing of proteins *in vitro* and *in vivo* are of vital importance for providing clinicians and scientists with sight and insight into the diagnostics and therapeutics of many a disease^1^,^2^,^3^,^4^. Recently, small-molecule fluorescence light-up probes that are capable of imaging specific proteins have been attracting increasing interest by virtue of their intrinsic advantages such as high sensitivity and large signal-to-noise ratio^5^,^6^,^7^,^8^. So far, several intelligent fluorescence quenching/activation mechanisms have been adopted to contrive small-molecule fluorescence light-up probes for protein detection (enzymes and non-enzymatic proteins), which include fluorescence resonance energy transfer (FRET)^9^, excited-state intramolecular proton transfer (ESIPT)^10^, photoinduced electron transfer (PET)^11^,^12^, restriction of intramolecular motions (RIM)^13^,^14^,^15^ and intramolecular charge transfer (ICT)^16^,^17^,^18^. In spite of these pioneering studies, successful examples that are able to selectively visualize proteins in live animals are rather limited^19^, which may be hampered by either poorly accessible to living organisms, low emission wavelength of fluorophores or small fluorescence turn-on ratio. As a consequence, new fluorescence light-up probes that can address the aforementioned limitations remain highly desirable.

In recent years, great interest has been focused on the design of small-molecule fluorescence light-up probes by employment of environment-sensitive molecules^16^,^17^,^18^,^19^. The fluorogens with environment-sensitive signature are often weakly fluorescent in polar media (*e.g*., water) because of the ICT-induced fluorescence quenching. However, the fluorogens could alter to show strong fluorescence when located in hydrophobic environment, which significantly reduces ICT between the fluorogen and polar media^20^,^21^. This unique fluorescence activation mechanism makes the environment-sensitive fluorogens an ideal material to fabricate fluorescence light-up probes with simple synthetic procedures, high sensitivity and large fluorescence turn-on ratio^5^,^19^. Nevertheless, the main obstacle in the *in vivo* application of environment-sensitive fluorogen-based light-up probes is that most of the currently available environment-sensitive fluorogens are low-wavelength emitters (<650 nm), which often suffer from relatively high biological background fluorescence and low tissue permeability^22^,^23^,^24^. Very recently, we have developed an environment-sensitive fluorogen, namely, 4,7-di(thiophen-2-yl)-2,1,3-benzothiadiazole (DBT) with strong ICT feature and far-red/near-infrared (FR/NIR) fluorescence^7^. The FR/NIR fluorescence window (650–900 nm)^25^,^26^ endows the DBT-based fluorescence light-up probes with great potential for *in vivo* bioimaging.

Lysosomal protein transmembrane 4 beta (LAPTM4B) is a vital biomarker for many solid tumours^27^,^28^,^29^. Detection and imaging of the emergence and localization of LAPTM4B proteins will significantly indicate the cancer occurrence, treatment efficacy and prognosis^15^,^30^,^31^. Therefore, exploration of small-molecule fluorescence light-up probes for LAPTM4B protein sensing is particularly valuable. In this contribution, a specific LAPTM4B protein detecting light-up probe with FR/NIR fluorescence was rationally designed and prepared *via* incorporation of DBT with a specific LAPTM4B-binding peptide IHGHHIISVG^15^. The peptide sequence EEG was also utilized as a hydrophilic linker between DBT and IHGHHIISVG to improve the water-solubility of the resultant probe (DBT-2EEGIHGHHIISVG, Fig. 1A). DBT-2EEGIHGHHIISVG is found to selectively light up LAPTM4B proteins in solution and cancer cells. Furthermore, the *in vivo* bioimaging study using a tumour-bearing mouse model reveals that DBT-2EEGIHGHHIISVG can also serve as an FR/NIR fluorescence light-up probe to selectively visualize the LAPTM4B protein-expressed tumour. To the best of our knowledge, this study reports the first example of fluorescence light-up probe for *in vivo* LAPTM4B imaging, which may inspire more exciting *in vivo* use probes in the future research.

## Results

### Synthesis and characterization of DBT-2EEGIHGHHIISVG

**Figure 1:**
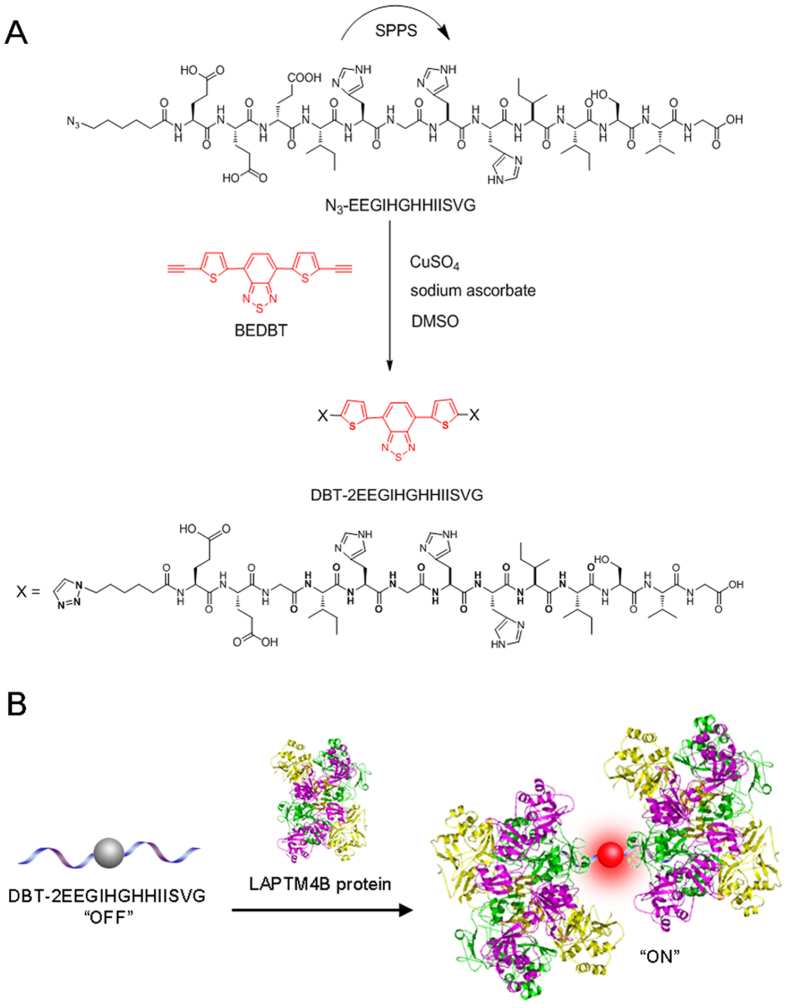
Synthetic route and proposed principle of the probe. (**A**) The synthetic route to probe DBT-2EEGIHGHHIISVG. (**B**) Schematic illustration of proposed principle of DBT-2EEGIHGHHIISVG in LAPTM4B protein detection.

The synthetic route toward DBT-2EEGIHGHHIISVG is depicted in Fig. 1A. The azide-functionalized EEGIHGHHIISVG (N_3_-EEGIHGHHIISVG) was firstly synthesized *via* standard solid-phase Fmoc peptide chemistry. DBT-2EEGIHGHHIISVG was then synthesized through “click” chemistry between peptides of N_3_-EEGIHGHHIISVG and bisethynyl-bearing DBT. N_3_-EEGIHGHHIISVG and DBT-2EEGIHGHHIISVG were characterized by ^1^H NMR, HPLC, LC-MS and HRMS (Supplementary Figs S1–S6) to verify the purity and identity. The UV-vis absorption spectrum of DBT-2EEGIHGHHIISVG shows a peak centred at around 490 nm (Supplementary Fig. S7), which pretty matches the 488 nm laser excitation of confocal laser scanning microscopy and the 523 nm excitation filter (ranging from 503 to 548 nm) of Maestro *in vivo* imaging system.

### Selective detection of LAPTM4B proteins in solution

**Figure 2:**
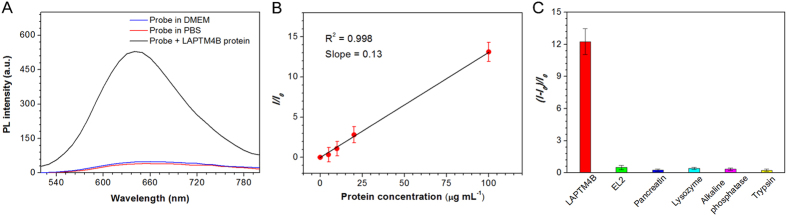
Specific fluorescence light-up toward LAPTM4B protein. (**A**) Photoluminescence spectra of DBT-2EEGIHGHHIISVG in PBS and cell culture medium as well as the probe after treatment with 100 μg mL^−1^ of LAPTM4B protein. (**B**) Plot of *I*/*I*_0_ *versus* LAPTM4B protein concentration. *I* and *I*_0_ are the fluorescence intensities of DBT-2EEGIHGHHIISVG in the presence and absence of LAPTM4B proteins, respectively. Data are expressed as mean ± SD (n = 3). (**C**) Plot of (*I*–*I*_0_)/*I*_0_ *versus* a variety of proteins and EL2. *I* and *I*_0_ are the fluorescence intensities at protein/EL2 concentrations of 100 and 0 μg mL^−1^, respectively. Data are expressed as mean ± SD (n = 3). [probe] = 10 μM and *λ*_ex _= 490 nm for (**A–C**).

The photoluminescence spectra of DBT-2EEGIHGHHIISVG in phosphate buffered saline (PBS) buffer and in cell culture medium (DMEM) are displayed in Fig. 2A. The probe (10 μM) is very weakly emissive in the above-mentioned media, suggesting the capability of DBT-2EEGIHGHHIISVG as a light-up probe to be utilized in complex biological environments. Furthermore, we have also demonstrated that unlike pure DBT, DBT-2EEGIHGHHIISVG does not interact with the cell membrane phospholipid thanks to its good water solubility (Supplementary Fig. S8). Noteworthy, upon incubation with LAPTM4B proteins, the specific binding of the probe with two LAPTM4B proteins would occur, leading to self-assembled complexes, which offers the DBT with hydrophobic microenvironment (Fig. 1B). As shown in Fig. 2A, after incubation with 100 μg mL^−1^ of LAPTM4B protein, the DBT-2EEGIHGHHIISVG fluorescence significantly turns on for about 13 times with maximum emission wavelength at around 640 nm. This should be attributed to the decreased ICT effect between DBT and polar media water by virtue of the hydrophobic microenvironment providing by the self-assembled complexes. The fluorescence life-time of DBT-2EEGIHGHHIISVG in the presence of LAPTM4B proteins is 1.9 times longer than that of the probe itself, revealing the change of probe microenvironment after binding with the proteins. As displayed in Fig. 2B, plot of fluorescence enhancement fold (*I*/*I*_0_) of DBT-2EEGIHGHHIISVG after treatment with LAPTM4B proteins as a function of LAPTM4B concentration gives a good linear line. The limit of detection (LOD) for DBT-2EEGIHGHHIISVG is then determined to be 2.4 μg mL^−1^ based on the slope in Fig. 2B *via* a 3-sigma method^32^.

To manifest that DBT-2EEGIHGHHIISVG is uniquely responsible for LAPTM4B protein, the probe was also incubated with a variety of other proteins, which include pancreatin, lysozyme, alkaline phosphatise, and trypsin. As shown in Fig. 2C, no obvious probe fluorescence activation can be seen upon treatments with these control proteins, suggesting the superb selectivity of our probe. It is also important to note that EL2 (PYRDDVMSVN; MW: 1194.5) has been reported as the hydrophilic extracellular loop of LAPTM4B protein, which is able to specifically bind to the peptide ligand IHGHHIISVG^15^,^30^. Actually, EL2 is the recognition site of IHGHHIISVG toward LAPTM4B protein. However, in our case, EL2 cannot switch on the fluorescence of DBT-2EEGIHGHHIISVG even at the concentration of 100 μg mL^−1^ (Fig. 2C). This is quite different from the results reported by Zhang and co-workers^15^,^30^. They developed a fluorescence light-up probe with aggregation-induced emission (AIE) characteristics for LAPTM4B protein detection, which was obtained by covalently coupling of an AIE fluorogen (AIEgen) and IHGHHIISVG. The fluorescence of AIEgen-IHGHHIISVG is able to be switched on upon addition of EL2, because their interaction could effectively restrict the intramolecular rotations of phenyl rings in AIEgens. However, it is obvious that the binding between the two small molecules of DBT-2EEGIHGHHIISVG and EL2 could not efficiently lead to assembled complexes that can provide DBT with hydrophobic microenvironment. Therefore, due to the different fluorescence activation mechanism, DBT-2EEGIHGHHIISVG is specific for bioimaging of LAPTM4B protein with large molecular weight.

### Targeted imaging of LAPTM4B proteins in cancer cells

**Figure 3:**
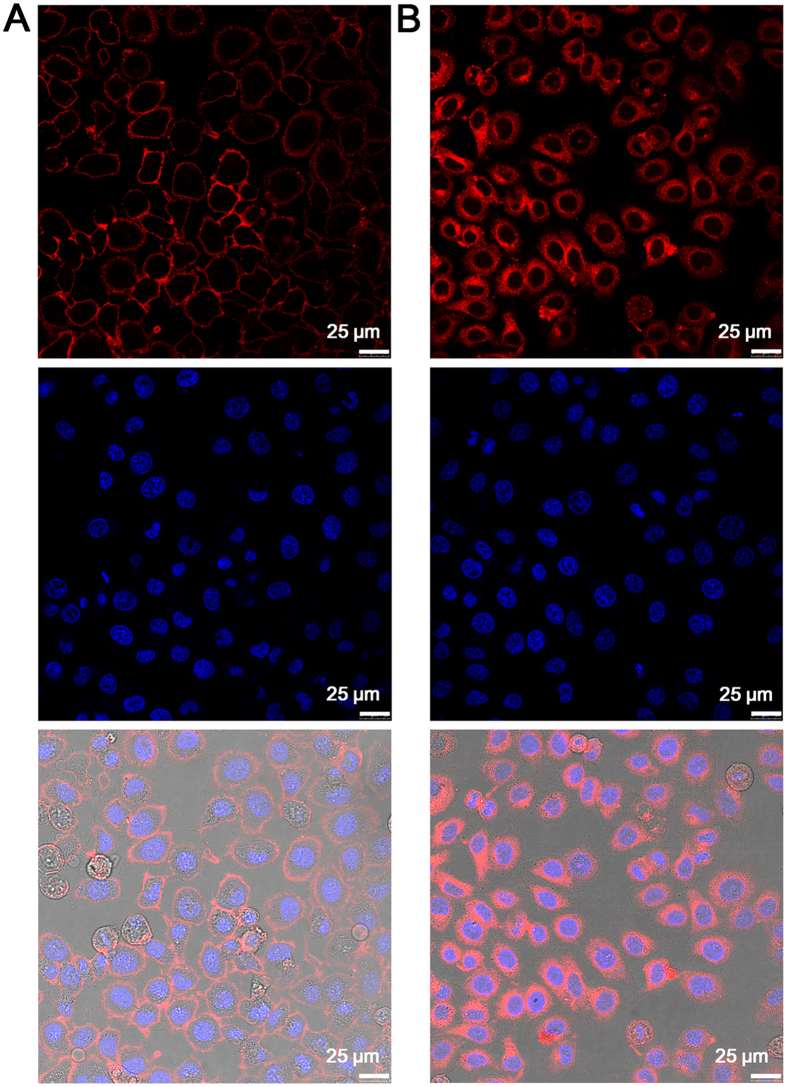
Targeted LAPTM4B protein imaging in HepG2 cancer cells. Confocal images of HepG2 cancer cells after incubation with DBT-2EEGIHGHHIISVG at (**A**) 0 °C and (**B**) 37 °C. The cell nuclei were stained by DAPI.

The utility of DBT-2EEGIHGHHIISVG in targeted imaging of LAPTM4B protein in cancer cells was investigated with confocal laser scanning microscopy. Human hepatocelluar carcinoma cells (HepG2) that are overexpressed LAPTM4B proteins were employed^15^,^30^. The HepG2 cancer cells were incubated with DBT-2EEGIHGHHIISVG (10 μM) on ice for 90 min, followed by imaging by confocal microscopy with 488 nm laser excitation and fluorescence signal collection above 650 nm. Fig. 3A shows the confocal images of DBT-2EEGIHGHHIISVG–treated HepG2 cancer cells at 0 °C. It is found that intense fluorescence signals are observed on the surface of the cancer cells, which is consistent with Zhang’s results^15^. This verifies that DBT-2EEGIHGHHIISVG specifically binds to LAPTM4B proteins on the membranes of HepG2 cancer cells. It is noted that by contrast, no detectable autofluorescence is observed from the cells without probe treatment under the same imaging condition (Supplementary Fig. S9).

**Figure 4:**
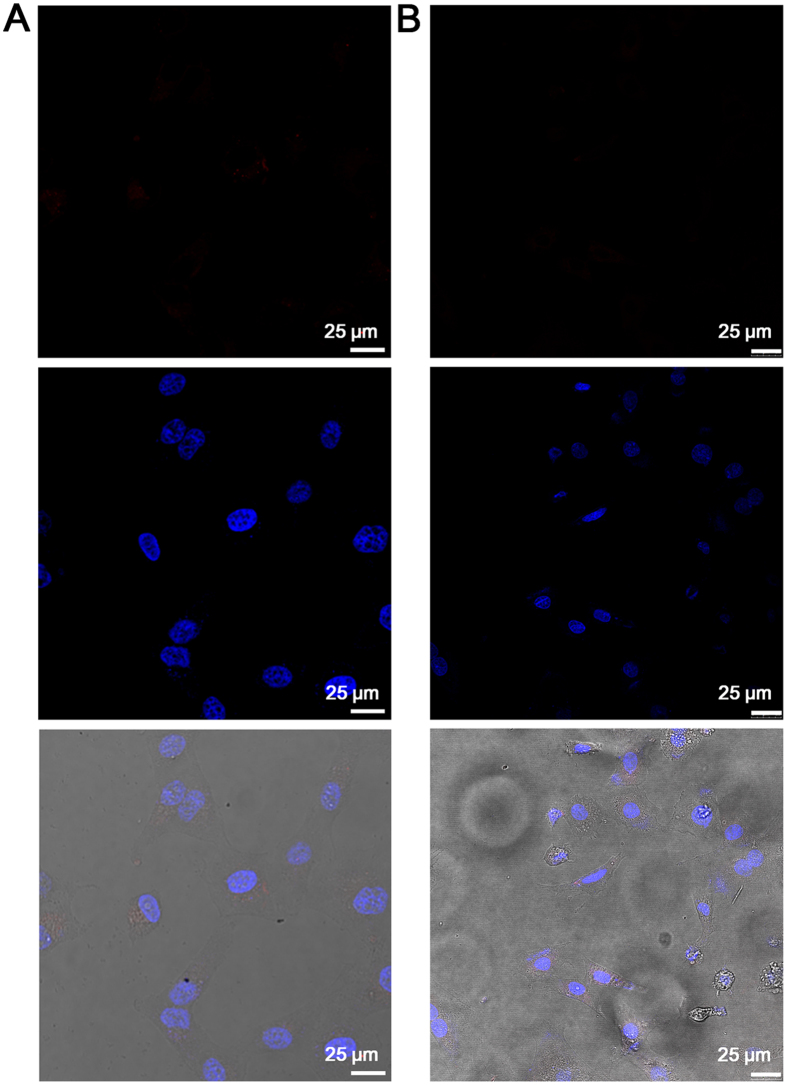
Cellular imaging in normal cells. Confocal images of (**A**) hepatic L02 cells and (**B**) smooth muscle cells after incubation with DBT-2EEGIHGHHIISVG for 90 min. The cell nuclei were stained by DAPI.

**Figure 5:**
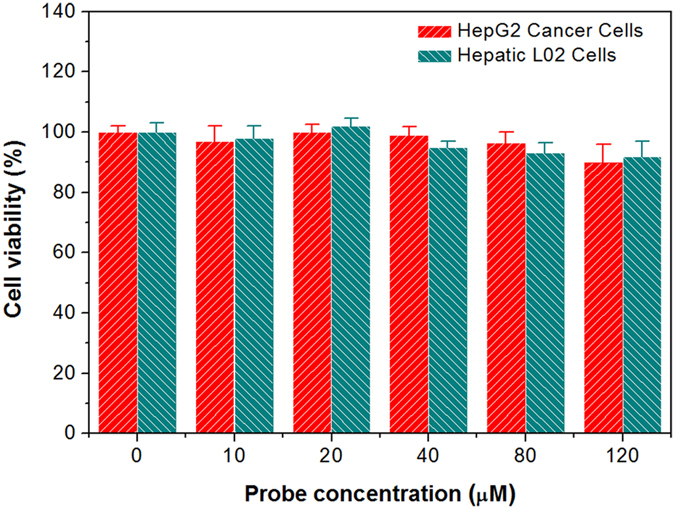
Cytotoxicity study. Metabolic viability of HepG2 cancer cells and hepatic L02 cells after incubation with DBT-2EEGIHGHHIISVG at different concentrations for 48 h.

In addition, DBT-2EEGIHGHHIISVG was also used to incubate with HepG2 cancer cells at 37 °C. As shown in Fig. 3B, obvious red fluorescence can be seen in both the membrane and cytoplasm of HepG2 cancer cells, which is further confirmed by the 3D confocal image (Supplementary Fig. S10). The competitive binding experiment *via* pre-treatment with IHGHHIISVG peptides verifies the specific binding interaction of the peptide ligand in the probe and LAPTM4B protein (Supplementary Fig. S11). These results reveal the cellular internalization of the probes through their specific binding with LAPTM4B proteins, which may also indicate that DBT-2EEGIHGHHIISVG is able to track the intracellular movement of LAPTM4B proteins. Furthermore, hepatic L02 cells and human smooth muscle cells were used as normal cell lines to verify the selective LAPTM4B protein imaging of our probe. After incubation of hepatic L02 cells or smooth muscle cells with DBT-2EEGIHGHHIISVG (10 μM) for 90 min, as displayed in Fig. 4, nearly negligible fluorescence signal can be detected in both the normal cells, which should be due to the very low expression of LAPTM4B proteins in these two cell lines. In comparison, HepG2 cancer cells and L02 cells were also incubated with pure DBT (10 μM) at 37 °C for 90 min, respectively. It is noted that the experimental condition for pure DBT is the same as that for DBT-2EEGIHGHHIISVG. As shown in Supplementary Fig. S12, similarly intense fluorescence signals are observed in pure DBT-stained HepG2 cells and L02 cells, revealing that pure DBT is not able to differentiate cancer cells (overexpressed LAPTM4B) and normal cells (low LAPTM4B expression) like DBT-2EEGIHGHHIISVG does. This result also verify the role and necessity of the peptide ligands in the probe. The cytotoxicities of DBT-2EEGIHGHHIISVG against HepG2 cancer cells and L02 cells were estimated by MTT assay. The data illustrated in Fig. 5 reveal that the cell viabilities remain more than 90% within 48 h even at the probe concentration of 120 μM (12 times higher than the concentration used for cellular imaging), implying that DBT-2EEGIHGHHIISVG is safe for both detected cancer cells and normal cells.

### Visualization of LAPTM4B protein-expressed tumours in live mice

**Figure 6:**
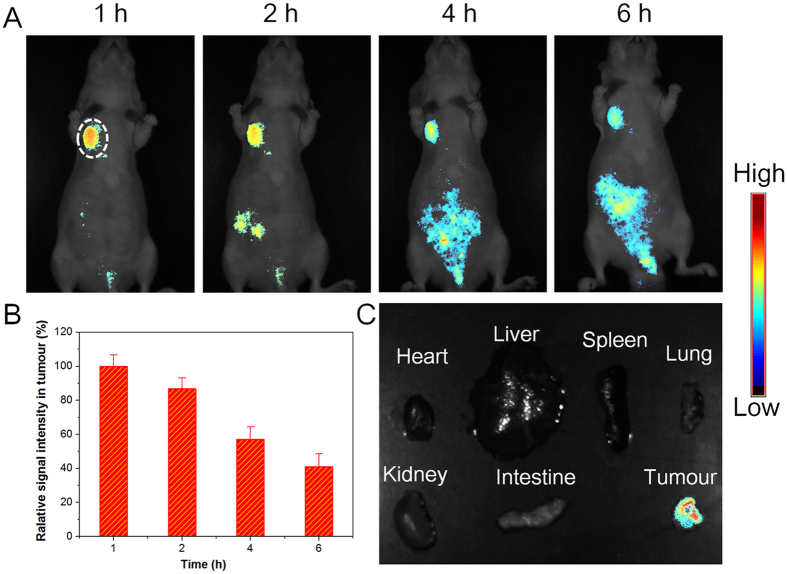
Imaging of LAPTM4B protein-expressed tumours in live mice. (**A**) Time-dependent non-invasive *in vivo* fluorescence images of HepG2 tumour-bearing nude mice after intravenous injection of DBT-2EEGIHGHHIISVG. The white circle indicates tumour site. (**B**) Semiquantitative analysis of the relative signal intensity in tumour tissue. (**C**) *Ex vivo* fluorescence image of various tissues from mice after intravenous injection of DBT-2EEGIHGHHIISVG for 1 h.

The application of DBT-2EEGIHGHHIISVG in *in vivo* imaging of LAPTM4B protein-expressed tumour tissues was next studied using non-invasive live animal fluorescence imaging technique^33^. In this experiment, HepG2 cancer cells were subcutaneously inoculated into the right axillae of the nude mice to establish the tumour-bearing mouse model. Subsequently, the tumour-bearing mice were intravenously injected with DBT-2EEGIHGHHIISVG, which was followed by imaging with a Maestro *in vivo* fluorescence imaging system. Spectral unmixing was employed to remove the mouse autofluorescence using Maestro software (Supplementary Fig. S13). As displayed in Fig. 6A, after intravenous administration of the probes for 1 h, strong fluorescence can be detected at the tumour site, which clearly delineates the tumour shape. It is encouraging that at 1 h, only intense signal from tumour tissue can be observed, whereas negligible fluorescence in other normal tissues is detected. As the time elapses, the fluorescence intensity in tumour gradually decreases but remains to indicate the tumour tissues at 6 h (Fig. 6B). Moreover, obvious fluorescence signals clearly appear in the abdomen regions of mice after 4 h, suggesting that the probes may be rapidly excreted from the mouse body^34^,^35^. At 24 h post-administration, almost no probe fluorescence signals can be detected in tumour and normal organs (Supplementary Fig. S14), demonstrating that most of the probes can be cleared from the body within 24 h. The fast metabolism of our probes is quite different from some carbon-based biomaterials that showed long-term retention in normal organs^36^, which may imply the negligible *in vivo* side toxicity, meeting the requirement of bioimaging. Furthermore, at 1 h post intravenous injection of DBT-2EEGIHGHHIISVG, the HepG2 tumour-bearing mice were sacrificed and various tissues including heart, liver, spleen, lung, kidney, intestine, and tumour were collected for *ex vivo* imaging. As displayed in Fig. 6C, only tumour tissue exhibits bright fluorescence signal of probe, while there is almost no detectable probe signals in other normal organs, which agrees well with the non-invasive result at 1 h post-injection. The result in Fig. 6 indicates that DBT-2EEGIHGHHIISVG is capable of visualizing LAPTM4B protein-expressed tumours of live mice in a selective and high-contrast manner at about 1 h post intravenous injection. As a control, pure DBT was also intravenously injected into HepG2 tumour-bearing mice. At 1 h post-injection, both non-invasive *in vivo* fluorescence imaging and *ex vivo* imaging data reveal that besides tumour, intense fluorescence signals are also observed in normal organs such as liver, intestine, and kidney (Supplementary Fig. S15), suggesting that pure DBT fails to selectively image LAPTM4B protein-expressed tumour *in vivo*. This result also verifies the rationality of our probe design.

## Discussion

In summary, we report the design and synthesis of an FR/NIR fluorescence light-up probe that can specifically detect and image LAPTM4B proteins in HepG2 cancer cells and in HepG2 tumour-bearing live mice. Thanks to the ICT character of DBT and good water-solubility of peptide of EEGIHGHHIISVG, DBT-2EEGIHGHHIISVG emits weakly in aqueous solution; however, the probe changes to fluoresce when LAPTM4B proteins specifically bind to IHGHHIISVG of the probe, as the complexes of probe/proteins provide the DBT with hydrophobic microenvironment, significantly reducing its ICT effect with water. Cellular studies using HepG2 cancer cells, hepatic L02 cells and smooth muscle cells reveal that DBT-2EEGIHGHHIISVG is a safe probe for targeted LAPTM4B protein imaging in cancer cells. The *in vivo* bioimaging study also manifests that after 1 h post intravenous injection of DBT-2EEGIHGHHIISVG, the probe has the capacity in selective visualization of LAPTM4B protein-expressed tumour tissues in live mice.

## Methods

### Chemicals

Fmoc-OSu and other Fmoc-amino acids were purchased from GL Biochem (Shanghai, China). 2-Cl-trityl chloride resin (1.0–1.2 mmol g^−1^) was purchased from Nankai University Resin Co. Ltd. Human LAPTM4B full length protein was bought from Abcam. All the other starting materials were bought from Sigma-Aldrich. Chemical reagents and solvents were utilized as received from commercial sources. Bisethynyl-functionalized DBT was synthesized according to previous literature^7^.

### General methods

^1^H NMR spectra were recorded on a Bruker ARX 400. HPLC was carried out at a LUMTECH HPLC (Germany) system using a C_18_ RP column with MeOH (0.1% of TFA) and water (0.1% of TFA) as the eluents. LC-MS was conducted at the LCMS-20AD (Shimadzu) system. High resolution mass spectra (HR-MS) were received from VG ZAB-HS system (England). UV-vis spectra were measured on a Shimadzu UV-1700 spectrometer. Photoluminescence spectra were measured on a Perkin-Elmer LS 55 spectrofluorometer. Cellular imaging was performed by confocal laser scanning microscopy (CLSM, Zeiss LSM 410, Jena, Germany). Non-invasive *in vivo* fluorescence imaging was conducted on a Maestro EX system (CRi, Inc.).

### Synthesis of azide-functionalized EEGIHGHHIISVG

The azide-functionalized EEGIHGHHIISVG was synthesized by solid-phase 9-fluorenylmethoxycarbonyl (Fmoc) peptide synthesis (SPPS) using 2-chlorotrityl chloride resin and the corresponding N-Fmoc protected amino acids with side chains properly protected by a tert-butyl group. Azide caproic acid was synthesized according to a previous report^7^ and used as an amino acid. The growth of the peptide chain was according to the established Fmoc SPPS protocol. The product was purified *via* HPLC (79% yield). ^1^H NMR (400 MHz, DMSO-*d*_*6*_, ppm) *δ*: 8.94–9.00 (d, 3 H), 8.41–8.46 (m, 1 H), 8.27–8.36 (m, 3 H), 7.97–8.18 (m, 7 H), 7.87–7.93 (m, 1 H), 7.71–7.77 (d, *J *= 8.66Hz, 1 H), 7.30–7.37 (t, 3 H), 4.56–4.71 (m, 4 H), 4.41–4.45 (m, 1 H), 4.17–4.30 (m, 6 H), 4.10–4.14 (m, 1 H), 3.70–3.80 (m, 8 H), 3.04–3.12 (m, 3 H), 2.93–3.02 (m, 3 H), 2.54 (s, 8 H), 2.22–2.26 (m, 2 H), 2.10–2.17 (m, 2 H), 1.97–2.03 (m, 1 H), 1.68–1.79 (m, 4 H), 1.47–1.56 (m, 4 H), 1.24–1.33 (m, 3 H), 1.00–1.11 (m, 3 H), 0.71–0.89 (m, 24 H). MS: calcd M^+^ = 1523.65, obsvd 1/2(M + H)^+^ = 762.50.

### Synthesis of DBT-2EEGIHGHHIISVG

Azide-bearing EEGIHGHHIISVG (12 mg, 7.9 μmol) and bisethynyl-functionalized DBT (1 mg, 2.9 μmol) were dissolved in 2 mL of DMSO. After that, 50 μL of 0.1 M CuSO_4_ and 50 μL of 1 M sodium ascorbate aqueous solution were added to the mixture to initiate the click chemistry. The reaction was proceeded at room temperature under stirring for 16 h to yield the final product (80% yield), which was purified by HPLC. ^1^H NMR (400 MHz, DMSO-*d*_6_, ppm) *δ*: 8.85–8.92 (m, 6 H), 8.63 (s, 2 H), 8.26–8.36 (m, 9 H), 8.17–8.21 (m, 5 H), 8.00–8.07 (m, 6 H), 7.72–7.76 (m, 2 H), 7.55–7.58 (d, 2 H), 7.24–7.33 (m, 10 H), 7.13 (s, 4 H), 7.00 (s, 4 H), 4.60–4.66 (m, 6 H), 4.39–4.44 (m, 6 H), 4.19–4.31 (m, 14 H), 4.10–4.16 (m, 4 H), 3.67–3.84 (m, 24 H), 2.65–2.69 (m, 2 H), 2.21–2.31 (m, 8 H), 2.12–2.19 (m, 4 H), 1.98–2.03 (m, 2 H), 1.86–1.92 (m, 6 H), 1.69–1.76 (m, 6 H), 1.52–1.58 (m, 4 H), 1.22–1.32 (m, 11 H), 1.15–1.20 (m, 2 H), 1.00–1.11 (m, 7 H), 0.71–0.88 (m, 48 H). MS: calcd M^+^ = 3395.77, obsvd 1/3(M + 3H)^+^ = 1133.00.

### Cell culture

Human hepatocelluar carcinoma cells (HepG2), hepatic L02 cells and human smooth muscle cells were cultured in Dulbecco’s Modified Eagle’s Medium (DMEM) containing 10% fetal bovine serum and 1% penicillin-streptomycin at 37 °C in a humidified environment containing 5% CO_2_, respectively. Before experiments, the cells were pre-cultured until confluence was reached.

### Cellular imaging

HepG2 cancer cells were seeded and cultured in confocal imaging chambers and then the cells were divided into two groups. One was placed at 37 °C and the other was placed at 0 °C for 30 min. Subsequently, the cells were washed three times with 1× PBS buffer and then incubated with DBT-2EEGIHGHHIISVG (10 μM) in serum-free cell culture medium at 0 °C or 37 °C. After incubation for 90 min, the cells were washed three times with 1× PBS and fixed using 4% paraformaldehyde for 20 min at 0 °C. The cells were then stained with 4′,6-diamidino-2-phenylindole (DAPI) for 10 min at room temperature, which was followed by imaging with confocal laser scanning microscopy upon excitation at 488 nm and a collection of fluorescence signals above 650 nm. The cell imaging studies of hepatic L02 cells and smooth muscle cells was carried out following the same experimental procedure as that for HepG2 cancer cells.

### Cytotoxicity study

The cytotoxicities of DBT-2EEGIHGHHIISVG against HepG2 cancer cells and hepatic L02 cells were evaluated by 3-(4,5-dimethylthiazol-2-yl)-2,5-diphenyl tetrazolium bromide (MTT) assay. Briefly, HepG2 cancer cells or L02 cells were seeded in 96-well plates at an appropriate density of 5 × 10^3^ cells of each well. After adherence, the cells were exposed to the probe at a pre-determined range of concentrations at 37 °C. After incubation for 48 h, the cell culture medium was removed and the cells were washed twice with sterile 1× PBS. 100 μL of MTT dissolved in serum-free culture medium (0.5 mg/mL) was added into each well. After 4 h incubation, the MTT solution was removed cautiously and then 100 μL of DMSO was added into the wells, followed by gently shaking for 10 min. The absorbance of MTT at 570 nm was determined by the microplate reader (Genios Tecan). To dispel the absorption interference of the probe at 570 nm, the cells were also incubated with the same concentrations of DBT-2EEGIHGHHIISVG as the control. After 48 h incubation, the control cells were not treated with MTT and then the absorbance at 570 nm were monitored by microplate reader. The absorbance of MTT in sample well was determined by the differentiation between the absorbance of the sample well and that of the corresponding control well. Cell viability was expressed by the ratio of the absorbance of MTT in the sample wells to that of the cells incubated with culture medium only.

### Animals and tumour-bearing mouse model

All animal studies were performed in compliance with the guidelines set by Tianjin Committee of Use and Care of Laboratory Animals and the overall project protocols were approved by the Animal Ethics Committee of Nankai University. 8 weeks old male BALB/c nude mice (obtained from the Laboratory Animal Center of the Academy of Military Medical Sciences (Beijing, China)) were utilized to establish the tumour-bearing mouse model. In brief, the mice were subcutaneously inoculated in the right axillae with 1 × 10^7^ HepG2 cancer cells. When the tumours reached to 200–400 mm^3^, the mice were used for *in vivo* imaging study.

### Non-invasive *in vivo* fluorescence imaging

The HepG2 tumour-bearing mice were injected with 200 μL of DBT-2EEGIHGHHIISVG or pure DBT at 120 μM *via* the tail vein. Subsequently, scans were conducted with a Maestro EX *in vivo* fluorescence imaging system at designated time intervals after administration. When taking images, the mice were temporarily anesthetized with isoflurane and placed appropriately. The 523 nm excitation filter (ranging from 503 to 548 nm) was selected and spectral imaging from 560 nm to 900 nm (10 nm step) was performed with an exposure time of 200 ms for each image frame. Additionally, the HepG2 tumour-bearing mice intravenously injected with DBT-2EEGIHGHHIISVG or pure DBT were sacrificed at 1 h post-administration. Subsequently, the main organs and tumour tissue were isolated and imaged with Maestro system.

## Additional Information

**How to cite this article**: Chen, C. *et al*. Far-red/near-infrared fluorescence light-up probes for specific *in vitro* and *in vivo* imaging of a tumour-related protein. *Sci. Rep*. **6**, 23190; doi: 10.1038/srep23190 (2016).

## Supplementary Material

Supplementary Information
