# Supplementary material for: Far-red/near-infrared fluorescence light-up probes for specific in vitro and in vivo imaging of a tumour-related protein
Source: Sci Rep. 2016 Mar 17;6:23190. doi: 10.1038/srep23190 (PMC4794726; doi:10.1038/srep23190)
Supplement: Supplementary Information [file srep23190-s1.doc]

Supplementary Information

Far-red/near-infrared fluorescence light-up probes for specific *in vitro* and *in vivo* imaging of a tumour-related protein

Chao Chen^1^, Yongquan Hua^2^, Yawen Hu^1^, Yuan Fang^1^, Shenglu Ji^1^, Zhimou Yang^1^, Caiwen Ou^2^, Deling Kong^1^ & Dan Ding^1^

**Figures.**

**
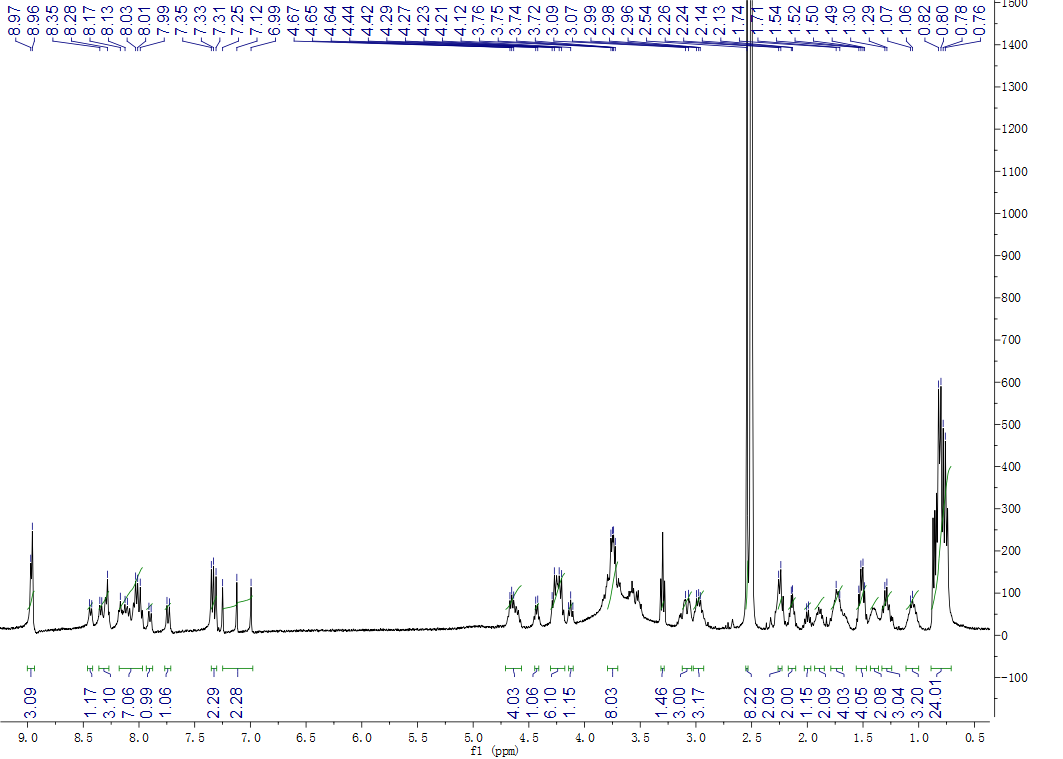
**

**Supplementary Figure S1** ^1^H NMR spectrum of N_3_-EEGIHGHHIISVG.

**Supplementary Figure S2** HPLC spectra and Mass spectrum of N_3_-EEGIHGHHIISVG (* stands for system peak).

**Supplementary Figure S3** HRMS spectrum of N_3_-EEGIHGHHIISVG.


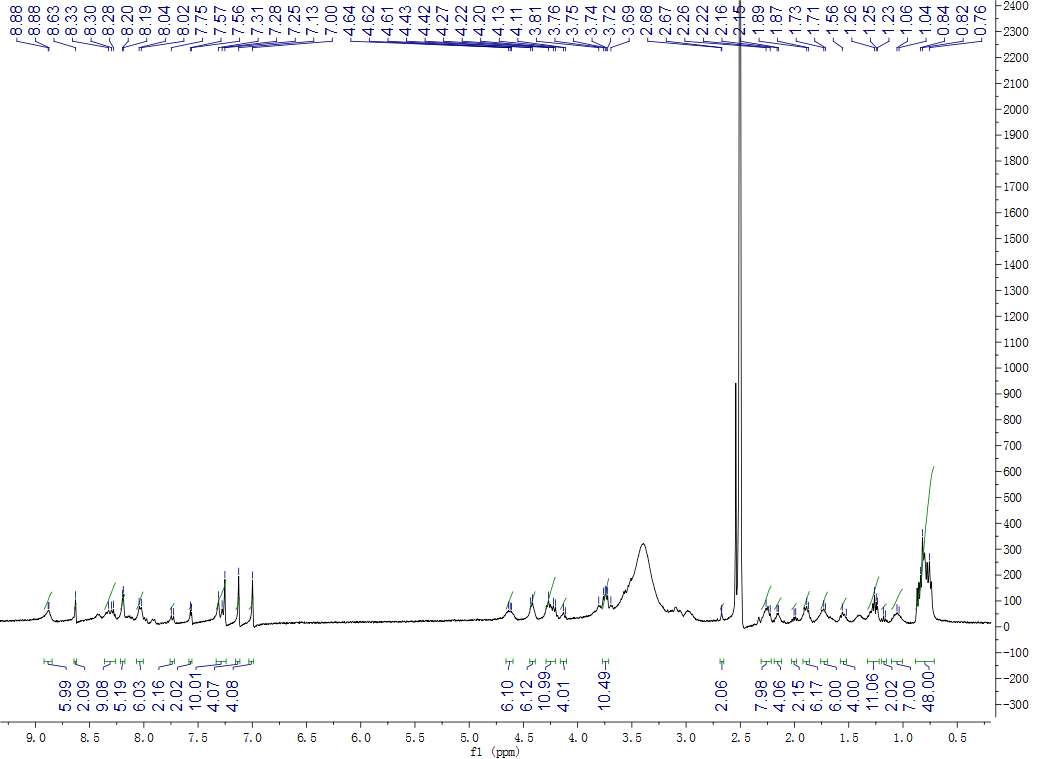


**Supplementary Figure S4** ^1^H NMR spectrum of DBT-2EEGIHGHHIISVG.

**Supplementary Figure S5** HPLC spectra and Mass spectrum of DBT-2EEGIHGHHIISVG (* stands for system peak).

**Supplementary Figure S6** HRMS spectrum of DBT-2EEGIHGHHIISVG.

**Supplementary Figure S7** UV-vis absorption spectrum of DBT-2EEGIHGHHIISVG in water.

**Supplementary Figure S8** PL intensities of DBT-2EEGIHGHHIISVG and pure DBT at 640 nm as a function of the added volume of cell membrane solution. In this experiment, 1×10^7^ 4T1 cancer cells were harvested, and 50 μL of extracting solution containing pure cell membrane was obtained (the cancer cell membrane derivation strategy is according to the literature "*Nano Lett.* **14**, 2181-2188 (2014)"). Subsequently, various volumes of the extract solution ranging from 0 to 15 μL were added to the aqueous solutions of probe (10 μM) and pure DBT (10 μM), respectively, followed by measurement of emission intensity at 640 nm. The result reveals that upon addition of cell membranes, the DBT-2EEGIHGHHIISVG fluorescence is still in "off" state, whereas the fluorescence of pure DBT significantly increases with the increase of cell membrane amount. This indicates that DBT-2EEGIHGHHIISVG would not interact with the cell membrane phospholipid thanks to its good water solubility and the hydrophobic interaction between pure DBT and membrane phospholipid provides DBT with hydrophobic microenvironment, leading to fluorescence enhancement. This result also indicates that the cell membrane phospholipid will not interfere with the LAPTM4B protein sensing of DBT-2EEGIHGHHIISVG.

**Supplementary Figure S9** Confocal image of HepG2 cancer cells without DBT-2EEGIHGHHIISVG incubation.


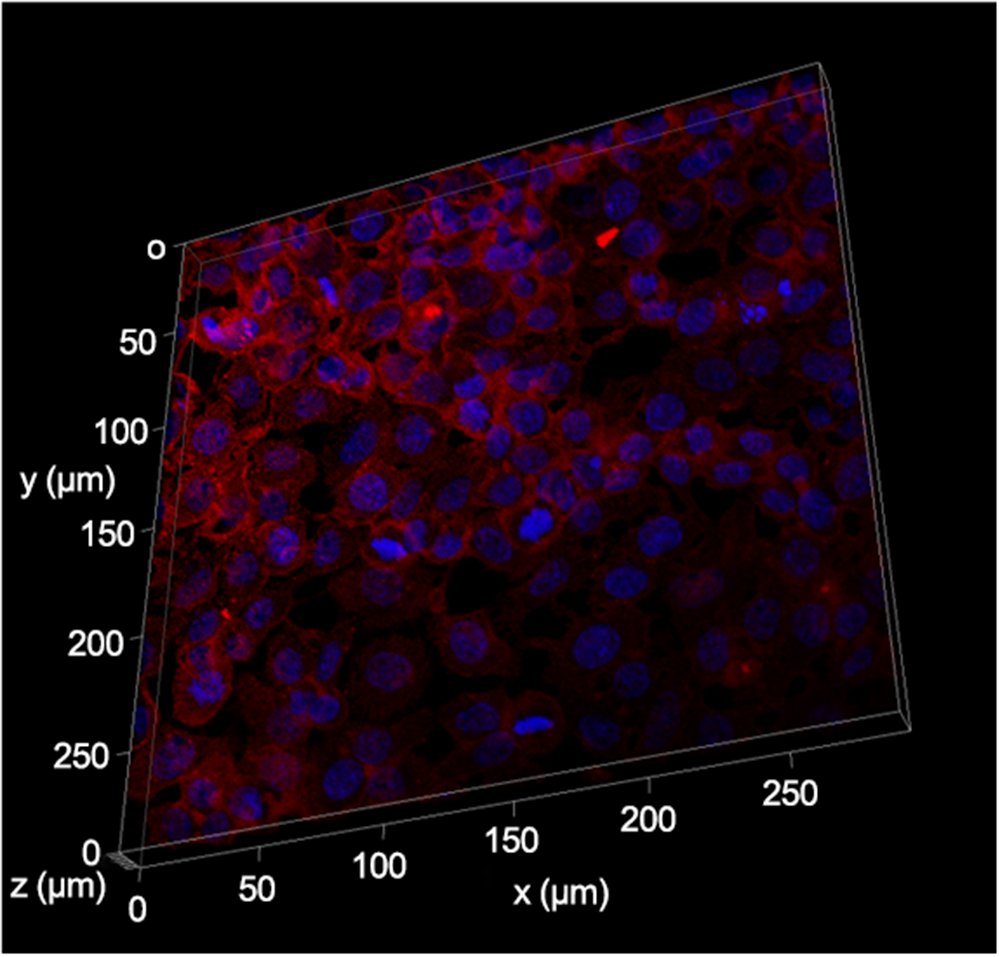


**Supplementary Figure S10** 3D confocal image of HepG2 cancer cells after incubated with DBT-2EEGIHGHHIISVG at 37 ^o^C.

**Supplementary Figure S11** Confocal image of DBT-2EEGIHGHHIISVG-incubated HepG2 cancer cells. The cells were pre-treated with IHGHHIISVG for 1 h.

**Supplementary Figure S12** Confocal images of (A) HepG2 cancer cells and (B) hepatic L02 cells after incubation with pure DBT at 37 ^o^C for 90 min. The cell nuclei were stained by DAPI. [DBT] = 10 μM.


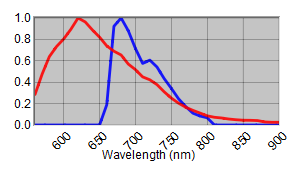


**Supplementary Figure S13** Spectra of DBT-2EEGIHGHHIISVG fluorescence (blue) and mouse autofluorescence (red) obtained by using Maestro *in vivo* fluorescence imaging system. Using spectral unmixing of Maestro software, the signals of DBT-2EEGIHGHHIISVG can be separated from the autofluorescence of mice.

**Supplementary Figure S14** *Ex vivo* fluorescence image of various tissues from HepG2 tumour-bearing mice after intravenous injection of DBT-2EEGIHGHHIISVG (120 μM) for 24 h.

**Supplementary Figure S15** (A) Non-invasive *in vivo* fluorescence imaging and (B) *ex vivo* fluorescence imaging of various tissues of HepG2 tumour-bearing mice after intravenous injection of pure DBT (120 μM) for 1 h. The white circle indicates tumour site.
